# Exploring the practice of Iranian adolescent females during menstruation and related beliefs: a qualitative study

**DOI:** 10.1186/s12889-024-18600-z

**Published:** 2024-04-24

**Authors:** Mojgan Mirghafourvand, Abbas Ebadi, Shayesteh Jahanfar, Fatemeh Khorashadizadeh, Shayesteh Shirzadi

**Affiliations:** 1 Social Determinants of Health Research Center, School of Nursing and Midwifery, Tabriz University of Medical Sciences, Tabriz, Iran; 2 Behavioral Sciences Research Centre, Nursing Faculty, Baqiyatallah University of Medical Sciences, Tehran, Iran; 3 Department of Public Health and Community Medicine, Tufts School of Medicine, Boston, USA; 4 Department of Epidemiology and Biostatistics, Neyshabur University of Medical Sciences, Neyshabur, Iran; 5 Healthy Ageing Research Centre, Neyshabur University of Medical Sciences, Neyshabur, Iran

**Keywords:** Menstruation, Adolescent, Female, Practice

## Abstract

**Background:**

Menstruation is a natural occurrence that women experience during their reproductive years and may encounter many years throughout their lifespan. Many adolescent females lack accurate knowledge about menstruation, so they may face issues from receiving incorrect information from unreliable sources. Our study aimed to investigate the practices and beliefs surrounding menstruation among Iranian adolescent females.

**Methods:**

This qualitative study was conducted using conventional content analysis. A purposeful sampling method was used to select 18 adolescent females from secondary and high schools located in the three regions of Neyshabur City-Iran. Data were collected through in-depth, semi-structured interviews.

**Results:**

Three main themes were extracted, consisting of lifestyle and related beliefs, lake of support, and awareness and information.

**Conclusions:**

misconceptions and wrong behaviors during menstruation indicate that the lake of knowledge an traditional factors influence adolescent girls’ health. The study provides the basis for intervention planning in this regard and different levels (individual, intrapersonal, health systems, and community).

## Background

Adolescence can be defined as the transitional period in life that spans from childhood to adulthood, comprisingthe ages of 10 to 19. It represents a distinctive phase in human growth and serves as a critical juncture for establishing the fundamental basis of good health [1]. In most countries of the world, especially developing countries, a significant part of the population is made up of adolescents [2]. Addressing adolescents’ health issues is beneficial not only for them but also for their families, society and the future generation, and special attention should be paid to their issues in the government policies of countries [3].

Adolescents experience rapid physical, cognitive and psychosocial growth. The most important change and evolution of this period, which is of particular importance and is considered as the turning point of adolescence, is puberty [4]. Although puberty and its issues are important in both adolescent girls and boys, it seems that paying attention to adolescent girls is more important compared to boys due to many physiological, reproductive health, cultural and social reasons [5].

One of the unique experiences of girls’ puberty is menstruation. Menstruation is a natural phenomenon of reproductive years [6] that women are faced to it during many years of their lifespan [7]. Menarche is a critical milestone in a girl’s life [8] and unlike other puberty changes that are slow and continuous, it has a sudden onset, so that it stays in the minds of adolescents girls and can be remembered easily [9]. Menstrual health is vital and an integral part of the health of adolescent girls and provides health for their adulthood [10]. Menstruation regularly and often disastrously stir ups their physical, mental, and social well-being of girls and women around the world and affects adversely on daily life and activities [11]. Most of the girls do not have proper knowledge about changes during puberty and healthy behaviors during menstruation and they face problems due to getting incorrect information from unreliable sources. In many cases, girls get their information from their mothers, unfortunately, mothers also do not have enough information in this field [12].

Based on the results of studies, adolescent girls do not have proper practice during the menstruation. They may change and restrict their diet [13], social performance [14] bathing [15] and religious functions [14, 16]. The lack of proper education and the avoidance of discussing about the changes during puberty and issues related to menstruation are another challenges of adolescent’s girl. Lack of accurate information and misconceptions regarding menstruation result in unhealthy behaviors [11]. Poor menstrual hygiene and unhealthy behaviors can face adolescent’s girl to health problems such as reproductive tract infections and also reduce the quality of life [6, 17]. People’s practice often stems from their beliefs [18]. Qualitative studies can provide an in-depth understanding of phenomena or experiences and are effective to capture beliefs, opinions, and behaviors of different groups of people [19]. It is necessary to perform qualitative studies to develop the health interventions they need, and improve the health behaviors [20].

Since, the menstrual practices can be influenced by various factors, and engaging in unhealthy behaviors during menstruation can have negative consequences for adolescent girls, by understanding adolescent female’s beliefs and their practice related to menstruation, we can establish necessary plans for appropriate effective interventions. This study aimed to investigate Iranian adolescent females’ menstrual practices and beliefs.

## Methods

### Study design, setting and participants

The present qualitative study was conducted using a conventional content analysis approach between February and July 2022 in Neyshabur city, Iran. Participants were selected using purposeful sampling method from six government secondary and six high schools located in the three regions of Neyshabur city with good, moderate and poor socio-economic status and maximum variation representing participants (Table 1). Sampling was continued until data saturation was achieved—wich is, no new information or codes were present in the data— which happened at the 14 th interview, and after achieving data saturation, other women were interviewed to supplement the data. A total of 18 adolesents femals participated in the interviews.

**Table 1:** Sociodemographic characteristics of participants

Variables		Ferecuncy
**Age (Years)**	12–14	9
	15–17	9
**Birth order**	1	6
	2	6
	≥ 3	6
**Mother’s job**	Housewife	14
	Employee	4
**Father’s job**	Employee	3
	Self-employed	15

Inclusion criteria were willingness to participate in the study, lack of physical or mental disability, and having at least three menstrual periods. The only exclusion criterion was unwilingness to continue in the study. All the participants voluntarily signed the informed consent form to participate in the study before the interviews. Also, in the case of minors, individuals younger than the age of 16, consent to participate was obtained from their parents or legal guardians. In addition, the school authority’s written permission was taken before the study. Ethical approval for the study protocol was provided by the Ethics Committee of Neyshabur University of Medical Sciences (ethics code: IR.NUMS.REC.1400.039).

### Data collection

Data collection was performed through individual, in-depth semi-structured interviews with the participants by an assistant professor of health education and promotion. Each interview was conducted through open-ended questions to explore the girls’ perceptions and opinions about issues related to menstruation such as diet, exercise, and bathing including “What do you think about exercising during your menstruation?“, Do you change your diet during your menstruation? And “What do you do to relieve menstrual pain?“. The subsequent questions asked were based on the participants’ responses. The interviews were done in schools. The interviews were conducted in Persian language and audio recorded. Each participant was interviewed in a session lasting approximately 30–60 min.

### Data analysis

Graneheim et al’s approach to qualitative content analysis [21, 22] was used to reflect the participants’ practices and opinions. The advantage of a conventional approach in qualitative content analysis, such as utilized in the current study, is the ability to gather data directly from study participants, while refraining from imposing preexisting categories and prior theoretical frameworks. In this method, the knowledge derived from content analysis is predicated upon the distinct perspectives of the participants and firmly grounded in textual data [23]. For this purpose, all interviews were transcribed verbatim and then carefully read several times in order to fully engage with the data and comprehend its central concepts. The analysis started by identifying meaning units that were drawn from the transcripts, then codes were generated inductively. For re-contextualization, the generated codes were compared and assigned to different subcategories based on differences and similarities. Subsequently, the subcategories were classified into categories through an additional interpretation adding the context to these designated categories (Table 2). The first author performed data analysis and other authors compared and revised the codes, subcategories, and categories. Data analysis continued until data saturation was achieved so that no other themes could be drawn.

**Table 2:** Examples of data analysis

Meaning units	Codes	Subcategory	Category
Peas and beans are flatulent and cause dysmenorrhea	Dysmenorrhoea due to flatulent food	Diet change	Lifestyle and related beliefs
I don’t eat yogurt when I’m on my menstruation because it makes my face blotchy	The formation of skin spots due to eating yogurt		
I avoid activities that cause heavy bleeding, such as running and jumping	Avoiding some heavy activities	Exercise and daily activities	
I should not lift heavy objects, this leads to back pain and increase menstrual bleeding	Lifting heavy objects causes heavy bleeding, Lifting heavy objects causes back pain		
I don’t take a bath during my menstruation	Don’t take a bath	Personal hygiene	
My uterus is open and water gets inside it, it makes me sick and I will get an infection.	Getting infection due to entering of water into the uterus		
Sometimes moms don’t understand their girls’ conditions	Lack of understanding of girls’ conditions by mothers	Not being understood	Lake of support
In my opinion, they should deal with them in such a way that the student is comfortable to talk to them	Lack of understanding of girls’ conditions by teachers		
I hide my period from my father	Hiding menstruation from the father	Shame and Embarrassment	
When I buy a sanitary napkin, I put it in black plastic bag so that it is not obvious and others cannot see it	Hiding sanitary napkins when shopping		
My mother does not have complete information	Incomplete information of the mother	Unawareness and Lack of Training	Awareness and Information
My mother says that you should not take a bath when you are on your menstruation because you will get an infection	Lake of knowledge		
I ask my questions of my mother	Mother as a source of information	Sources of Information	
If I have a question or need information about menstruation, I usually search on the Internet	Internet source of information		

### Data trustworthiness

In order to enhance the credibility and reliability of the data, efforts were made to establish a positive relationship between the interviewers and participants, and sufficient time was allotted for data collection. Furthermore, the interview transcripts and codes were shared with the participants to obtain their feedback and perspectives on the extracted meaning units to ensure accuracy and validity.In addition to the research team, the transcripts were also presented to a number of experts, as external observers, who were not involved in the study, so that they could examine the accuracy of the coding process.

## Results

In total, 208 codes and 7 subcategories were extracted, which were classified into 3 main categories including lifestyle and related beliefs, lake of suport, awareness and information (Table 3).

**Table 3:** Extracted main categories and subcategories

Main Category	Subcategory
Lifestyle and related beliefs	Diet change
	Exercise and daily activities
	Personal hygiene
Lake of support	Not being understood
	Shame and embarrassment
Awareness and information	Unawareness and lake training
	Sources of information

### Lifestyle and related beliefs

Most of the participants expressed that they changed or modified their lifestyle during menstruation. This category is comprised of three subcategories, diet, exercise and daily activities and personal hygiene.

#### Diet change

Most of participants believed that the diet should be changed and the consumption of some foods such as dairy, pickles and sour fruits, spicy foods, flatulence foods, fast food, foods with cooling effect on the body on Iranian traditional medicine and some vegetables should be restricted during menstruation or few days before beginning it. They believed the mentioned foods could cause or exacerbate menstrual pain or disrupt menstrual regularity or cycle. Other beliefs included the effect of some food on skin health for example pickles and spicy foods cause acne and pickles, tomato, yogurt, foods with a cold temperament and fish cause face stain. Some believed consuming foods with a cold temperament and pickles during menstruation will lead to infertility in adulthood. A girl said:

*“I don’t eat pickles or sour things like plum, leather (pestil), or foods with a cold temperament like rice, yogurt and milk, because of pain, pickles increase the pain and foods with a cold temperament reduce menstrual bleeding.” (Participant number 14, 16 years old)*.

*“ My sister says don’t eat watermelon and tomato, watermelon makes period smell worse, I think tomato also thickens period blood, I don’t know exactly.” (Participant number 1)*.

*“My mom says don’t eat yogurt, your face will be stained, or she won’t let me eat the potage, she says peas and beans are flatulent and cause dysmenorrhea. " (Participant number 7)*.

#### Exercise and daily activities

Most of the participants stated that they modified or restricted exercise, physical activity and some daily activities during menstruation. These activities included jumping, bodybuilding, and going to the gym or sports clubs and lifting heavy objects. They believed that these activities cause back pain, dysmenorrhea and increased menstrual bleeding.

*“I know, When I have menstruation, I should not lift heavy objects and do not exercise. These lead to back pain and increase menstrual bleeding.” (Participant number 6)*.

*“ During my period, I avoid activities that cause heavy bleeding, such as running and jumping.” (Participant number 1)*.

*“ I go to the gym and I walk for an hour a day, but I don’t go to the gym when I’m on my period, but I walk, like other days, walking is not a heavy exercise. " (Participant number 1)*.

#### Personal hygiene

We found conflicting beliefs among participants regarding bathing and washing the genital area during menstruation. The most of participants reported restricting bathing during menstruation or some reduced the bathing duration. Also, Some participants limite or reduce the frequency of washing their genital area. Some misconceptions including the effect of bathing on menstrual blood flow, entering water to uterine lead to infection, uterine cancer, or infertility and that bathing or washing genital area could worsen menstrual symptoms such as back pain, cramps, or heavy bleeding.

*“I don’t take a bath during my menstruation because my uterus is open and water gets inside it, it makes me sick and I will get an infection.” (Participant number 8)*.

*“My mother told me not to take a bath, it is dangerous, but I take a bath myself, because if I don’t take a bath, my body will get dirty, during this period, hygiene has a great impact on body’s health” (Participant number 10)*.

*“ I take a bath like the days before my period, but I take a bath standing up, because the floor of the bathroom may be contaminated and I may get sick. " (Participant number 2)*.

### Lake of support

This category comprised of tree subcategories, not being understood, bloody and stained clothes and shame and embarrassment.

#### Not being understood

Several participants expressed frustration that their families do not understand their mental health conditions, such as preferring to be alone. Additionally, some shared that their school staff did not prioritize their menstrual pain and sometimes ignored their needs. These participants felt that their school environment was not supportive, citing examples such as a lack of access to sanitary napkins for menstruating students.

*“Sometimes moms don’t understand their girls’ conditions, they don’t let them be in their own way. I like to be in my own way, I don’t want to be around anyone around at all, I like be alone but my mom does not understand me.” (Participant number 14)*.

*“One day I got my period at school, I didn’t have sanitary napkin, it wasn’t available in the school buffet, I had to borrow from the school janitor.” (Participant number 13)*.

#### Shame and embarrassment

Some participants felt shame of menstruation. They hide their menstruation from others especially from males. They were embarrassed to ask their questions about menstruation and complained that their mothers did not talk and discuss about of menstruation and its related issues with them. Another issue that girls found embarrassing was going to a store to purchase sanitary napkins, especially when the shopkeeper was male. Also, They stated that feel ashamed of blood stains and leakage on their clothing, which caused them to avoid social interactions.

*“I hide my period from my father, I don’t like him to know that I’m on my period, I am ashamed. Also, my mothers stress that I be careful not let father/brother notice that I am menstruating.” (Participant number 4)*.

*“I buy sanitary napkins from a female seller, if it’s a male seller, I don’t buy it. Also, it is important to me that I put it in black plastic so that it is not noticeable.” (Participant number 3)*.

*“When I’m menstruating, I do not feel comfortable when my father is at home, because I am constantly concerned that my clothes and my bed do not get bloody that it makes me ashamed.” (Participant number 12)*.

### Awareness and information

This category is comprised two subcategories (1), Unawareness and lack of training (2) Sources of information.

#### Unawareness and lack of training

The participants’ knowledge about the menstrual phenomenon and what was mentioned above, such as pain relief methods, personal hygiene (washing genital area and bathing), diet, physical activity, and exercise during menstruation, was inadequate or incomplete. They complained of poor training regarding puberty, menstruation and their related subjects by family, society and school. Also, they said that they were not familiar with menstruation, and that the most of time training and provided information by family, frinds and at school were partial, incomplete or incorrecte. They expressed frustration that they could not ask their family members questions freely about menstruation due to feelings of shame and embarrassment.

*“My mother told me, in the first three days of your menstruation, you should not take a bath at all, or if I go to the toilet, as my mother told me, you should not flush your genital with pressured water because the cervix is open, so that the water enters the uterus and lead to cancer.” (Participant number 6)*.

*“In my opinion, the educational classes about menstruation should be held in schools because I am not comfortable with my mother and I cannot ask her my questions easily.” (Participant number 18)*.

*“ I am comfortable with my mother, if I have a question, I ask her, but my mother does not have complete information, if she cannot answer my question, I will ask my sister.” (Participant number 3)*.

#### Sources of information

One of the prominent features of this study was the role of mothers and their influence on girls’ beliefs and performance. Traces of mothers were present in most of the girls’ statements and all that was reported in the study. In most of their statements, there was the phrase “my mother says”. At all, the participants identified their mothers and family members, such as sisters, as the most common sources of information about menstruation. They also mentioned getting information from friends, relatives, teachers, social networks, the Internet, books, and physicians.

*“I ask my questions of my mother, I am comfortable with her, I am not embarrassed.” (Participant number 8)*.

*“ I am ashamed to ask my questions to my mother, when we go to the health center, my mother is with me, so I cannot ask my questions to the health workers, I do not go anywhere alone, for this reason, if I have a question, I search on the Internet or ask my friends.” (Participant number 8)*.

## Discussion

This qualitative study aimed to explore how Iranian adolescent girls explain their menstrual practice and related beliefs. he results showed that despite the availability of information through books, media, social networks and the internet, etc., adolescent girls still had incorrect practices and wrong beliefs. Our findings highlight the influence of inadequate knowledge and misconceptions on wrong practices of Iranian adolescent girls regarding menstruation, as well as the influence of the environment and the people who surround them, especially mothers.

According to our findings, most participants changed their lifestyle during menstruation, including diet, exercise, daily activities, personal hygiene behaviors, etc. Other researchers have also reported restrictions on work activities, exercise or physical activity, attending social functions [24], bathing [25], washing genitalia area [26], participate in religious activities [27], and eating certain foods [28].

Consistent with our study, results of a study conducted in Iran showed that women and girls limit eating some foods (cold foods, sour foods, salty food, foods causing gas, dairy, citrus) during menstruation and even one week before it, as they believe it causes cramping, may have an effect on the sex of the child in the future, cause smelly menstrual secretions and decrease chance of future pregnancy [27]. There is this belief that certain foods can hasten or delay menstruation [29]. Also, change in appetite and food craving during the menstrual cycle has been reported that is consistent with our finding [30].

Participants’ preferences and beliefs regarding genital washing and bathing varied widely. There are prevalent misconceptions among girls about these practice during menstruation; for example, taking a bath during menstruation increases the flow of menstrual blood and intricacies during pregnancy [25] and, washing during menstruation impact blood flow [31]. In studies conducted in India, washing the genital area during menstruation varied from 30 to 94% [14, 26]. Unhealthy behaviors such as not bathing and washing the genitals [32] and not using sanitary pads [33] cause reproductive tract infections, and other vaginal diseases that adversely affect the health of girls and their future fertility. Genital infection was higher in those who took a bath in a sitting position or once or less per week and did not do genital cleaning [34, 35].

Menstruation is a topic surrounded by culture and menstrual practices are strongly influenced by cultural beliefs, unfortunately, most of these beliefs are wrong and rooted in unawareness [25]. In our study, adolescent girls considerd their family member especially their mothers, as appropriate sources of information and their influence from their mothers was evident in most of their beliefs and behaviors. Unfourtunetly, in the most of the time, the mothers themselves do not have correct knowlege or their information is insufficient [36, 37]. Although menstruation is a subject that is related to more than half of the world’s population, nearly all cultures are uncomfortably discussing it. Therefore, most girls experiencing their menarche without prior information and being unprepared for menarche.

Experience of physical and mental symptoms was reported by the participants during or before menstruation. In a survey of 42,879 women between the ages of 15 and 45, the reported symptoms included abdominal pain during period, heavy bleeding, headache, back pain, tiredness, and premenstrual psychological complaints [38]. These symptoms can affect on different aspects of life, such as attendance and performance in school, activities of daily living [39], and quality of life [40]. Menstrual symptoms even lead to a significant economic burdens, mainly due to work productivity loss. A study in Japan estimated that annual financial burden extrapolated to the Japanese female population was ∼8.6 billion Dollars [41].

Dysmenorrhea is a common menstrual problem that affects adolescent girls’ life [42]. It reported as the most common cause of short-term absence from school, and about 1 in 8 girls aged 14 to 20 years missing school or work due to dysmenorrhea [43]. In our study, most participants suffered from dysmenorrhoea and believed that dysmenorrhoea is inevitably part of the menstrual cycle. They were self–medicating and their preference for treatment was non-medical management rather than medical management. According to the evidence, methods such as rest, sleeping, heat, herbal drugs, hot drinks, or sports, and medicine (such as paracetamol ibuprofen, aspirin, or a spasmolytic, etc.) for managing pain have been reported [44]. Despite the high prevalence of dysmenorrhea, it is often underdiagnosed, inadequately treated, and normalized even by patients themselves, who may accept the symptoms as an inevitable response to menstruation. Women with menstrual pain should not be dismissed; thus, increasing public awareness of the recently available medical treatments can improve the overall burden of menstrual problems [45].

Participants expressed some concerns related to menstruation, including shame and embarrassment and stained and bloodied clothing not to be understood by others (such as their family members and teachers). Also, some of them stated sometimes there was not a supportive environment in some schools in terms of educating about menstruation and providing sanitary napkins. Unfortunately, most did not have adequate information about healthy and proper behaviors during menstruation and had wrong practices. In addition, they complained about the lack of appropriate and adequate education about menstruation and lack of information and stated that they needed education about menstruation.

Although menstruation is a normal biological process and more than half of the world’s population experience it, nevertheless there is many concerns and challenges about it. Still, people are not comfortable to talk and discuss about it, and female are still ashamed of staining their clothing, menstrual products, going to outlet and buying the sanitary napkin and hide their menstruation especially from men [46]. Many young adults and adolescents feel unprepared for menstruation [47].

Menstruation can be both physically and emotionally distressing. Therefore, instrumental, informational, and emotional support can help to manage related concerns and issues [47]. Emotional support from parents, friends, and other important persons can reduce the psychological and emotional problems associated with it [48]. Adolescent girls need information about hygiene products, pain management, medication, and other health-related information; they need informational support in detail about menstruation and related issues. It reported when information is provided as an in-depth explanation about the menstruation process would be more effective [23]. Consistent with our study, previous studies indicated mothers as the most important information source regarding menstruation, followed by peers and school nurses [44]. Mothers’ attitudes toward menstruation shape the menstrual information girls receive, which, if inadequate, might negatively affect their daughters’ practice of menstruation [46]. Based on the results of studies, mothers did not want to openly discuss about menstruation and were secretive and uncommunicative on this issue, wich reflects traditional feminine gender roles [48, 49]. In this kind of circumstances, social media platforms can use as a tool for addressing knowledge gaps, breaking the silence around menstruation, and building a caring community among participants. The online communities and social networks focused on menstrual health can serve as platforms for collective learning, knowledge sharing, knowledge co-creation, and emotional-relational support, empowerment, solidarity, and the potential for social change [23].

The weak knowledge of mothers and even teachers regarding menstruation and adherence to some traditions can result in unfair practices in adolescent girls and damage their health. Some restrictions (such as avoidance of eating some foods, bathing, washing genital area and, etc.) can hurt physical health; some (such as hiding menstruation, embarrassed of bloody and stained clothes, and restrictions on participation in social interactions and events) can affect the mental health or social inclusion. Mothers, teachers and, etc. themselves should prepare for the task of educating their adolescent girls about menstruation. The function of the mothers in menstrual education is particularly crucial because girls may learn related behaviors primarily from them. Reproductive and menstrual health and hygiene should include in the school curriculum. Teachers hardly talk and guide the girls about menstrual health and hygiene. There should be orientation programs for teachers and school counselors. Holding educational sessions on menstruation can help correct these false beliefs and misconceptions among mothers and their children. Interventions that are practical, sustainable, and culturally acceptable must inform the community, especially parents and their adolescent girls, to empower them in their transition to womanhood. Local Health Committees should be involved in education and awareness programs. A specific handbook and website should be developed on menstruation and menstrual hygiene management, clarifying myths, misconceptions, and taboos. Therefore, socializing messages, support from parents, educators, and healthcare providers, and other interpersonal communication about menstruation are needed to promote healthy behaviors in adolescent girls.

This study had a number of limitations. First, generalization of the results of this study, considering its qualitative approach, should be done cautiously that could be considered as a limitation. Although qualitative studies do not make for the generalization of the results, they might be necessary for those who are willing to use the results of these studies. Second, we did not triangulate the results of our study with quantitative approaches for this component. Conversely, the strengths of the study were selecting participants with maximum variations, guidance and supervision of experts, and external reviewing, the effort was to increase the accuracy and transferability of the data.

## Conclusion

The results show that despite the availability of information through books, media, internet, etc., adolescent girls still had incorrect practices and wrong beliefs. Most the participants get their information from their family members, especially their mother and relatives, while unfortunately, based on what explored from the participants, they did not have enough information and proper practice.

## Data Availability

All data generated or analysed during this study are included in this published article.
